# Allostatic Load as an Insight into the Psychological Burden after Primary Treatment in Women with Breast Cancer: Influence of Physical Side Effects and Pain Perception

**DOI:** 10.3390/jcm11082144

**Published:** 2022-04-12

**Authors:** Azzurra Irelli, Jessica Ranieri, Maria Maddalena Sirufo, Francesca De Pietro, Pamela Casalena, Lia Ginaldi, Katia Cannita, Dina Di Giacomo

**Affiliations:** 1 Medical Oncology Unit, Department of Oncology, Azienda Sanitaria Locale di Teramo, 64100 Teramo, Italy; azzurra.irelli@hotmail.it (A.I.); pamela.casalena@aslteramo.it (P.C.); kcannita@gmail.com (K.C.); 2 Laboratory of Clinical Psychology and Psychoncology, Department of Life, Health and Environmental Sciences, University of L’Aquila, 67100 L’Aquila, Italy; dina.digiacomo@univaq.it; 3 Department of Life, Health and Environmental Sciences, University of L’Aquila, 67100 L’Aquila, Italy; maddalena.sirufo@gmail.com (M.M.S.); fra722@hotmail.it (F.D.P.); lia.ginaldi@univaq.it (L.G.); 4 Center for the Diagnosis and Treatment of Osteoporosis, Allergy and Clinical Immunology Unit, Azienda Sanitaria Locale di Teramo, 64100 Teramo, Italy

**Keywords:** breast cancer, allostatic load, psychological distress, physical impairment, mental health

## Abstract

Breast cancer (BC) diagnosis and treatment have become a cumulative long-standing chronic disease impairment, causing stress and turning into an allostatic load (AL) framework. This study aimed to investigate the relationship between physical issues and mental health in patients with BC after medical treatment. We conducted an observational study of 61 female patients with BC, and clinical and psychological markers have been detected. We conducted descriptive statistics, ANOVA analyses, correlations, and mediation analyses to verify the effect of the comorbidity index on psychological dimensions. The findings showed high levels of distress and moderate pain, and 32.8% of the patients showed moderate physical impairment. Significant effects of “age” and “physical issues” were found. The adult group reported a higher incidence of physical issues, and the group of patients reporting moderate physical impairment seemed more depressed than patients with mild physical issues. Finally, the comorbidity condition mediated the presence of signs of depression. Patients with BC seemed to experience negative emotions related to comorbidities associated with compromised activities of daily living. Our findings highlighted allostatic overload as a predictive framework to better understand the mental health of women with BC diagnoses to tailor effective psychological treatments for enhanced recovery.

## 1. Introduction

In recent years, breast cancer (BC) has come to be considered a chronic disease. Survival after BC diagnosis is positively changing because of efficient healthcare, such as early screening and early BC diagnosis, higher sensitivity of screening tools, and clinical innovation in surgical and pharmacological treatments [1,2].

A cancer diagnosis can be highly stressful and potentially traumatic [3,4,5,6,7]. Experiencing intense symptoms, multiple cancer-related side effects, fear of disease progression, as well as recidivism, fear of death, and worry about job loss can increase emotional stress and chronic psychological distress, including anxiety, anger, and depression [8,9,10,11,12]. Receiving primary oncological treatments could be a particularly traumatic experience due to the impact on patients’ self-perception (e.g., chemotherapy-induced alopecia, mastectomy/lumpectomy, changes in weight, changes to skin and nails texture), reduced intimacy with their partner, breakdown of social relationships, and a demanding psychological burden, implying the need for the highest adaptive behaviour to the new condition from a new life perspective [13,14,15].

Following cancer diagnosis, surgery, chemotherapy, and multiple cancer-related complications, many cancer patients suffer from chronic stress; the related impact is on endocrine and behavioural reactions that are regulated by neurochemical systems; the subsequent physical and mental dynamics could lead to inflammation, ischaemia-reperfusion injury, sympathetic nervous system activation, and increased cytokine release. Negative psychological dimensions have been associated with lower white blood cell activity, reduced antibody levels, and increased stress hormone responses [15]. Abercrombie’s study [16] showed that women with BC display elevated basal cortisol levels and decreased acute cortisol reactivity compared with healthy controls. Patients with cancer experiencing chronic stress are often burdened with a variety of adverse symptoms, including neuroendocrine dysregulation, anxiety, depression, anger, other mental health disorders, and sleep disturbances [17,18,19]. If left untreated, these disorders can lead to reduced quality of life and poor compliance with cancer treatments, with repercussions on overall survival [20,21]. 

From this perspective, the allostatic load (AL) framework could be considered to manage long-term activation of stress response systems in women with BC, which refers to the cumulative biological burden exerted on the body’s systems due to repeated adaptation to stressors over time. AL is intended to measure cumulative physiological stress across major regulatory systems (e.g., endocrine, metabolic, cardiovascular, and immune), resulting from dysregulated stress hormones in the hypothalamic–pituitary–adrenal axis [22,23,24,25,26,27]. In particular, the clinical diagnosis of allostatic overload could be helpful in a comprehensive approach that seeks to understand how the interactions among genetics, mind, body, behaviour, and environment can determine low psychological and mental responses to treatment.

Chronic psychological distress increases AL in patients with BC, promoting poorer health outcomes and disease progression. Ruini’s study [28] investigated the relationship between the post-traumatic growth and AL in different stressful experiences (cancer vs. major life events). The study showed that allostatic overload was more common among cancer survivors (52%) than among healthy controls (33%).

As highlighted above, several studies have focused on BC mental health issues that detect negative psychological aspects and emotional dimensions. However, few studies have analysed mental health management in patients with BC, highlighting the allostatic factors that determine the adaptation process in survivorship. In clinical practice, assessing AL features might serve as a suitable indicator of well-being deterioration and multiple adverse health outcomes. Monitoring the vulnerability of patients and predicting the onset of psychiatric comorbidity seem useful for mental health care. The hypothesis of this study is that women with BC experience allostatic overload. 

The present study aimed to preliminarily determine the mental health of women with BC using an allostatic load framework. The primary endpoint of the study was to examine the physical and psychological dimensions of women with BC after analysing the physical and psychological imbalance in integrated care. The secondary endpoint was to analyse the relationship between physical impairment and comorbidity as an allostatic overload factor in psychological adaptation to oncological treatments.

## 2. Materials and Methods

### 2.1. Ethical Approval

This study was approved by the institutional review board of the University of L’Aquila, Italy (Prot. No. 16372/2019). Written informed consent was obtained from each participant and the study adhered to the Declaration of Helsinki [29].

### 2.2. Participants

Participants were composed of *n* = 61 female patients with a mean age of 63.9 ± 11.1 years, who have a BC diagnosis and are receiving chemotherapy treatment. Eligible participants were approached to be enrolled in the study at the Medical Oncology Division (Director Dott Katia Cannita) of G. Mazzini Hospital, ASL2 Abruzzo in Teramo, Italy. We contacted 70 eligible patients, of whom 61 provided informed consent. Demographic and clinical characteristics of the participants are shown in Table 1. 

The inclusion criteria were as follows: (a) BC diagnosis in the last 6–9 months; (b) finalised chemotherapy treatment; (c) histopathological BC diagnosis of positive and HER2-negative hormone receptors, and (d) hormone therapy-associated Prolia and vitamin D treatment.

Exclusion criteria were: (a) diagnosis of rheumatic diseases (e.g., rheumatoid arthritis, osteoarthritis, fibromyalgia); (b) osteoporosis diagnosis; (c) no signs of recidivism in the time period (0–6 months) after primary treatment (drop-out); (d) metastases; (e) dental treatments against antiresorptive therapies; (f) psychiatric disorders, and (g) mood- and sleep-modifying medications.

### 2.3. Measures

#### 2.3.1. Sociodemographic Variables

Two types of participant information were also collected. First, demographic data were collected through participants’ self-reports. Second, clinical data were obtained from participants’ medical records regarding BC stage, treatments, and therapies.

#### 2.3.2. Psychological Measurement

The psychological battery was composed of two self-assessment screening tools that measure emotional traits (depression, anxiety, and psychological distress), detailed as follows. 

Hospital Anxiety and Depression Scale (HADS) [30]. This self-report scale measures symptoms of depression and anxiety over the past week. It is composed of 14 questions (7 items for depression (HADS-D) and 7 items for anxiety (HADS-A)), and responses are indicated on a 4-point Likert-type scale with scores ranging from 0 (not at all) to 3 (a lot), with the highest scores indicating more severe anxiety and depression (score from 0 to 7, no symptoms; score from 8 to 10, mild symptoms of depression and/or anxiety; score from 11 to 14, moderate symptoms of depression and/or anxiety; score from 15 to 21, severe symptoms of depression and/or anxiety).

National Comprehensive Cancer Network Distress Thermometer (DT) [31]. This is a visual analogue scale that can be immediately interpreted to rule out high levels of distress in patients with cancer. The DT score ranges from 0 (no distress) to 10 (severe distress), and must be completed by the patient, taking into account their mood over the last week. A cut-off score of 3 was considered optimal for ruling out significant distress.

Numerical Rating Scale (NRS) [32]. The NRS measures the perceived pain intensity with a rating from 0 (no pain) to 10 (most severe pain). Mild pain is reflected by NRS scores of 1–3, moderate pain by NRS scores of 4–6, and severe pain by NRS scores of 7–10.

Cumulative Illness Rating Scale (CIRS) [33]. This scale assesses physical impairment. The scale classifies comorbidities into 14 systems and rates them according to severity, from 0 to 4 (0 for no impairment to the specific organ/system; 1 for mild impairment that does not interfere with normal activity, in which treatment may or may not be required and prognosis is excellent; 2 for moderate impairment which interferes with normal activity, where treatment is necessary and prognosis is good; 3 for severe impairment that is disabling, in which treatment is urgently needed and prognosis is guarded, 4 is extremely severe, where the impairment is life-threatening and treatment is urgent, or prognosis is poor). The CIRS was used to evaluate the comorbidity index (the number of categories rated as moderate or severe). 

#### 2.3.3. Procedure

The medical staff of Medical Oncology identified patients with BC between July 2019 and January 2020. Written informed consent was obtained from all the participants at the time of enrolment. Trained clinical psychologists (blinded to the aim of the study) conducted the psychological assessments in a quiet, dedicated room. The duration of the evaluations was 20 min. Participants underwent further psychological evaluation post-primary oncological treatments (8–12 weeks after diagnosis). Data were collected anonymously. 

#### 2.3.4. Study Design

We conducted an observational single-centre study to investigate the allostatic load on mental health among patients with BC after primary oncological treatments; we wanted to analyse the impact of physical issues as the allostatic overload factor of patients psychologically recovering from post-medical treatment.

#### 2.3.5. Statistical Analyses

Descriptive statistics for measures were calculated to analyse the emotional characteristics of the target. ANOVA analyses (followed by Tukey’s post hoc analyses) were conducted to detect the statistical significance of the overall differences across the psychological variables, by comorbidity index and age. Pearson’s r correlation was then applied. A mediation analysis was performed to verify the effect of the comorbidity/disability index on depression.

The Jamovi Stat was used for the statistical analyses [34]. The level of significance was set to α < 0.05.

## 3. Results

Raw scores and standard deviations of participants’ performance are reported in Table 2. 

Descriptive analyses by test scoring showed no signs of anxiety (HADS-A score < 8), depression (HADS-D score < 8), high levels of distress (DT ≥ 4), or moderate pain intensity (NRS ≥ 5). Considering the frequency values of physical issues, 67.2% presented mild physical issues, and 32.8% of patients showed moderate physical impairment.

First, we analysed the effect of age on emotional variables (anxiety, depression, and distress), pain intensity, and physical impairment. Participants were divided into two groups according to median age (65 years): young group (Yg) and adult group (Ag). One-way ANOVA (3 × 2) was conducted to compare the performance of the young and adult groups in the psychological tests (HADS-D, HADS-A, and DT). The statistical analysis showed no significant differences.

**Figure 1:**
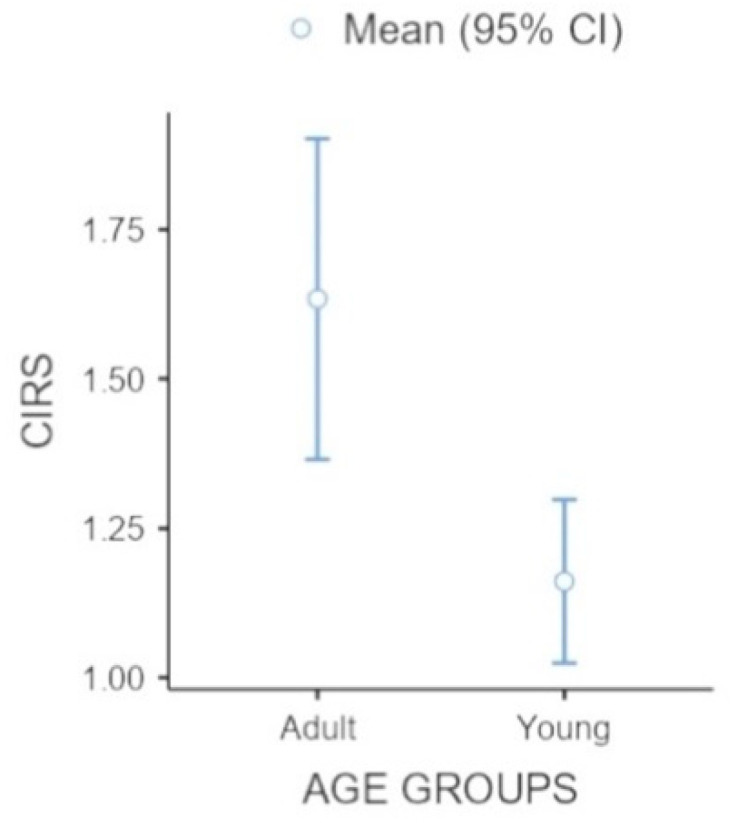
Representation of CIRS index by young and adult groups.

We wanted to verify the influence of age on physical side effects and pain perception. A one-way ANOVA was conducted to compare the performance of Yg and Ag groups in CIRS and NRS tests (Table 3). Analyses showed a significant difference in the CIRS test (F(10,2) = 43.3; η = 0.4; *p* = 0.001); post-hoc analysis evidenced that the adult group reported a higher incidence of physical issues (see Figure 1). No differences emerged between the groups according to the NRS index (pain intensity).

**Figure 2:**
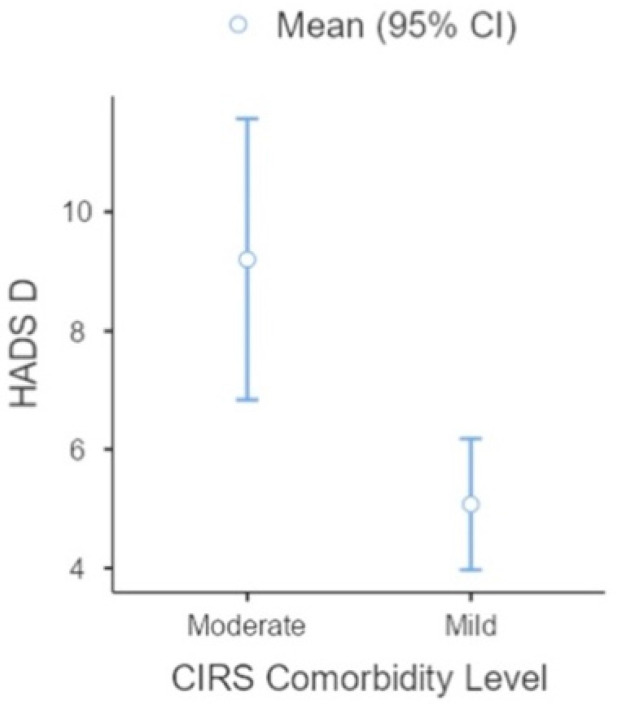
Representation of degree of depression distributed by moderate and mild CIRS indexes.

Subsequently, by categorising the sample on the physical side-effect indices, we distributed the participants into two CIRS index groups: (1) mild physical issues, in which participants had a CIRS score of 1; (2) moderate physical impairment where participants had a CIRS score of 2. We conducted an ANOVA (3 × 2) analysis comparing psychological dimensions (HADS-A, HADS-D, and DT) by CIRS group (mild and moderate physical impairment). We found a significant effect on the HADS-D variable (F(13,9) = 59; η = 3.7; *p* < 0.001), whereas no difference was observed in HADS-A or DT (see Table 4). The group of patients reporting moderate physical impairment seemed to be more depressed than those with mild physical issues (see Figure 2).

Finally, we processed the ANOVA (4 × 2) comparing psychological dimensions (HADS-A, HADS-D, DT, and CIRS) and surgical intervention (lumpectomy vs. mastectomy), and no significant differences emerged. 

**Figure 3:**
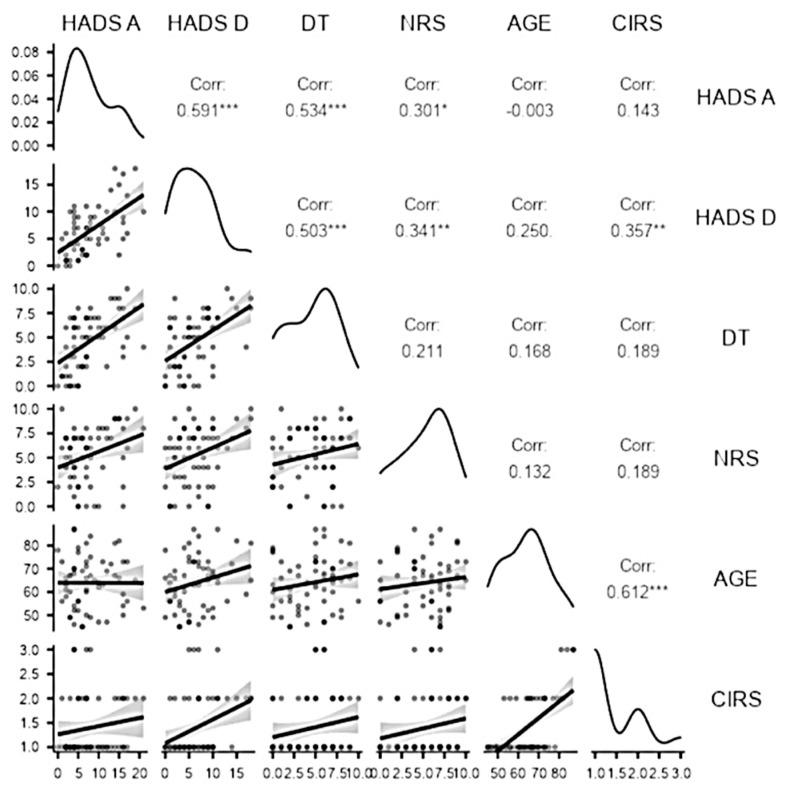
Plot for Pearson r correlations between HADS, DT, NRS, and CIRS scores for the sample. Note: * = *p* < 0.05; ** = *p* < 0.01; *** = *p* < 0.001.

Furthermore, we examined the relationship between physical issues (CIRS) and psychological dimensions (HADS-D, HADS-A, and DT) and pain intensity (NRS). We conducted Pearson’s correlation analysis, and the results are summarised in Figure 3. As expected, distress (DT) was positively correlated with anxiety (HADS-A) (*r* = 0.53; *p* < 0.001) and depression (HADS-D) (*r* = 0.50; *p* < 0.001); pain intensity (NRS) was significantly correlated with anxiety (HADS-A) (*r* = 0.30; *p* = 0.01) and depression (HADS-D) (*r* = 0.34; *p* = 0.007); finally, CIRS was positively correlated with depression (HADS-D) (*r* = 0.35; *p* = 0.005).

**Figure 4:**
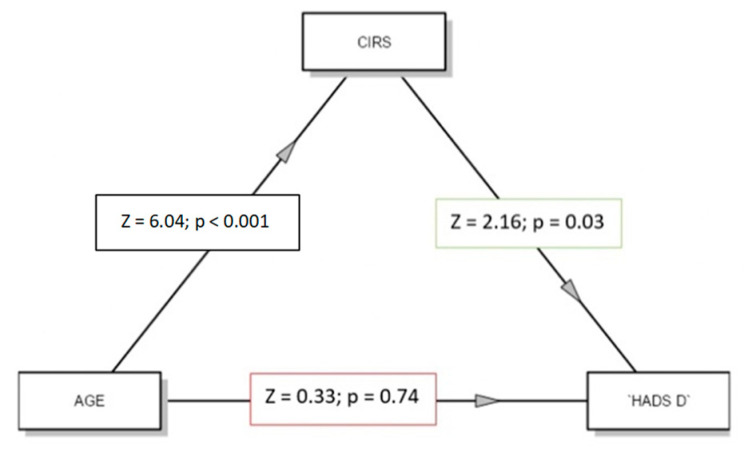
Representation through model diagram of mediation effect of CIRS.

Finally, we examined the relationship between physical issues and depressive factors. We conducted a GLM analysis to test the direct and indirect influence (mediation effect) of the comorbidity index (CIRS) on depression. In the mediation model (see Figure 4), age was the independent variable, comorbidity (CIRS) was the mediator variable, and depression (HADS-D) was the dependent variable, and we wanted to verify the direct influence of age on depression as well as the indirect effect of comorbidity. As shown in Figure 4, the comorbidity index mediated depression (SE = 0.03, β = 0.19, z = 2.03, *p* = 0.004), with no direct effect of age on depression. 

## 4. Discussion

The study aimed to examine the psychological dimensions of women with a BC diagnosis and their relationship with the physical side-effects of medical treatment, as well the pain perception. Our scope was to investigate the characteristics of allostatic load of women with BC who had undergone primary oncological treatments; the relationship among physical, perceived pain, and psychological factors have been investigated to explore the impact of medical treatments to comorbidity.

According to the literature, allostatic overload in women with BC should be better investigated [35,36,37,38,39] as the framework for efficient health management in survivorship favouring emotional resilience. As highlighted in some research protocols, women with BC experience negative emotions and transform that into personal growth [40,41,42,43,44]; the improvement of mental health into the AL framework is becoming an emerging topic. According to the AL framework, and confirming the emotional regulation model in cancer experience [45], our study confirmed the positive frame of mind of women with BC after primary oncological treatments, evidencing a strong ability to manage the negative emotions through an emotional regulation process producing adaptive responses, as well as mental flexibility in clinical settings. Our findings showed no signs of psychopathological conditions. 

Overall, our findings demonstrated the effect of physical issues on emotion regulation in women with BC: high comorbidity appeared predictive of low clinical adherence to pharmacological treatment in survivorship; moreover, high comorbidity was related to the development of depressive signs, regardless of age. 

Regarding aging and comorbidity factors, older women with BC after primary oncological treatments might show significant functional impairments, as well as secondary illness, providing evidence for polypharmacological therapies. According to some studies, this might complicate treatment outcomes and exacerbate the vulnerability of older patients with BC [46,47,48]; our finding appears to confirm the vulnerability of patients to aging in terms of increasing side effects, physical impairments, and comorbidities. According to Zhao [49], the risk of elevated AL tends to increase with age over time. 

The mediating role of comorbidity factors on the development of depression created a psychological burden for patients with BC towards the design of AL as a screening tool for high-risk health outcomes in cancer. In fact, patients with BC with several physical impairments seemed to be associated with high depression levels, indicating relevant AL. 

The present study aimed to contribute to the investigation of health implications for well-being and quality of life in patients with BC by exploiting the utility of specific allostatic overload operationalisations in the scientific setting of biobehavioral medicine research and practice. Our findings provide details on the life course dynamics of allostatic load-related processes in women with BC. Practical outcomes for the AL framework in cancer survivorship should address enhanced daily living and adherence to pharmacological treatments, side effects of self-perception, and overall comorbidity effects, through a multidisciplinary patient-centred approach. The relevance of emotional management in a patient-centred approach could favour patient engagement in dealing with the rapidly changing physical and mental effects: the active behaviour in the perception of one’s own psychological dimensions (and needs) from the patient could contribute to improved survivorship, based on reducing the allostatic overload for an enhanced quality of life. 

There are some limitations to the study: (a) typology of measurements, (b) limited biomarkers, and (c) sampling study design. First, the research protocol was based on the detection of emotional traits by screening measures, and future research should adopt structured psychological measurements to draw the emotional regulation and psychopathological dimensions of patients with BC. 

Second, the biomarkers considered in the study were based on comorbidity variables as well as subjective values of perceived pain index; more extensive data (as well as more types of biological markers) could be acquired to boost the significance of the findings, such as the applicability of the AL framework. 

Finally, the sampling strategy could in the future be based on better stratification of survival timing in the longitudinal study design. 

## 5. Conclusions

Our study offers a new perspective regarding the negative psychological dimensions to physical impairment: patients with BC seemed to suffer negative emotions related to disability and comorbidity associated with compromised activity in daily life. Physical impairment appeared to be predictive of negative emotional adaptation.

This is the first study to show significant relationships between physical impairment and comorbidity as allostatic overload factors in psychological adaptation to oncological treatments. Physical impairment as an allostatic overload factor could be routinely incorporated as a screening tool or health outcome in BC patients to provide tailored effective psychological support. 

## Figures and Tables

**Table 1:** Demographic and clinical data of participants.

Sample (*n* = 61)	N pts ^1^ (%)
Education	
Did not graduate	40 (65.5)
Graduated high school	10 (16.3)
Bachelor’s degree	11 (18.0)
Occupation	
Housewife	11 (18.0)
Self-employed	4 (6.5)
Employed	11 (18.0)
Retired	35 (57.3)
Relationship status	
Single	5 (8.1)
Married	34 (55.7)
Divorced	12 (19.6)
Widow	10 (16.3)
Treatments ^2^	
Lumpectomy	40 (65.5)
Mastectomy	21 (34.4)
Chemotherapy	28 (45.9)
Hormonal therapy	32 (52.4)
Radiotherapy	41 (67.2)

Note: ^1^ pts = patients; ^2^ treatments are not mutually exclusive.

**Table 2:** Raw scores of psychological testing by age groups.

Tests	^µ^ Yg (*n* = 31) M ± SD	^£^ Ag (*n* = 30) M ± SD	Sample (*n* = 61) M ± SD
HADS			
A *	7.4 ± 5.2	8.2 ± 5.2	7.8 ± 5.2
D ^¥^	5.3 ± 4	7.5 ± 4.6	6.4 ± 4.4
NRS	4.7 ± 2.8	5.8 ± 2.7	5.2 ± 2.8
CIRS	1.1 ± 0.3	1.6 ± 0.7	1.3 ± 0.6
DT	4.0 ± 2.7	5.2 ± 2.7	4.6 ± 2.8

Note: ^µ^ Yg = Young group; ^£^ Ag = Adult group; * HADS-A = Anxiety; ^¥^ HADS-D = Depression; NRS = Numerical Rating Scale; CIRS = Cumulative Illness Rating Scale; DT = Distress Thermometer.

**Table 3:** Group description data and one-way ANOVA statistical analysis.

Test	Age Groups	*n*	Mean	SD	F	*p*
CIRS	Ag	30	1.63	0.71	10.46	0.002
	Yg	31	1.16	0.37		

**Table 4:** One-way ANOVA on Depression data by CIRS indexes.

Test	CIRS Indexes	*n*	Mean	SD	F	*p*
HAD-D	Mild	41	9.20	5.05	13.89	0.0001
	Moderate	20	5.07	3.48		

## Data Availability

The data presented in this study are available upon request from the corresponding author.
